# Editorial: Functional materials with charge transfer properties and their application in photoelectric devices

**DOI:** 10.3389/fchem.2022.1094264

**Published:** 2022-11-23

**Authors:** Meng Zheng, Teresa Gatti, Yue Liu, Yongtao Qu

**Affiliations:** ^1^ R&D Center of Polymer Materials, Qingdao Haiwan Science and Technology Industry Research Institute Co., Ltd. (HWSTI), Qingdao Haiwan Chemistry Co., Ltd. (QHCC), Qingdao, China; ^2^ Institute of Physical Chemistry, Justus Liebig University, Giessen, Germany; ^3^ Center for Materials Research, Justus Liebig University, Giessen, Germany; ^4^ College of Material Science Engineer, Liaoning Technical University, Fuxin, China; ^5^ Department of Mathematics, Physics and Electrical Engineering, Northumbria University, Newcastle Upon Tyne, United Kingdom

**Keywords:** charge transfer properties, structure-property relationships, applications, photoelectric devices, device performance

## Introduction

Charge transfer is an extensively studied issue as this property enables molecular components to manipulate and tune the properties of materials. This is achieved through a systematic variation of the molecular components, which allow for a molecular-level control of the structural-property *via* an arrangement of the functional molecular components into a defined architecture by charge generation and extraction.

In our topic “*Functional Materials with Charge Transfer Properties and Their Application in Photoelectric Devices*”, we received 14 interesting articles which concerned more on perspectives and new developments of functional material with charge transfer properties, leading to good performance in different type of devices, such as fluorescence sensor, organic field effect transistors, solar cells, lithium storage, nanocomposite supercapacitor, etc. Strategies of molecular design and simulation to improve device performance were also discussed in this topic as well as some extensive research work relating to charge transfer properties.

## Charge transfer properties and device applications

Hou et al. reviewed the application of biological sensing based on dicyanomethylene-4H-pyran (DCM) derivatives with intramolecular charge transfer (ICT) properties. The luminescence mechanism of DCM probes mainly depends on the ICT properties. By regulating the ICT process, the DCM probes have been constructed to detect the ions, reactive oxygen species (ROS), and biological macromolecules in cells. DCM derivatives modified with fluorescence-quenching group have shown typical off–on characteristics.

Meanwhile, Cai et al. designed and synthesized DCM-based derivatives, enable to fluorescence enhancement in solid state for visualization of latent fingerprints (LFP). In this study, the authors prepared DCM derivatives with strong emission in solid state by introducing Boc group, which provides strategies for the fluorescence enhancement of aggregation-caused quenching dyes in solid state. LFP fluorescent developers were prepared by blending Boc-PZ-DCM with montmorillonite (MMT). It showed that LFP can be clearly developed by dusting method with 3% dye content Boc-PZ-DCM/MMT developer. Boc-PZ-DCM/MMT developer for LFP reduced the content of fluorescent materials to 3%, greatly reducing the consumption of fluorescent materials and increasing the safety of LFP fluorescent developer.

Zhang et al. designed a pyrrolopyrrole-based aza-BODIPY (PPAB) small molecule for organic field-effect transistors based on thiophene-substituted diketopyrrolopyrrole (DPP). Due to large conjugated molecular skeleton, the PPAB units exhibited a broad absorption range in the visible and near-infrared regions, which enable it to be a new chromophore with electron-deficient ability. The OFETs constructed by PPAB as the semiconductor layer present a clear p-type behavior with a maximum electron mobility of 1.5 × 10^−3^ cm^2^ V^−1^ s^−1^, indicating that PPAB is a promising electron-deficient chromophore to construct semiconductors for OFETs.

Another application of OFETs based on small molecular design was taken by Dai et al. A novel alternating donor–acceptor polymer PQ1 is designed and synthesized by palladium-catalyzed Stille coupling. Polymer PQ1 presents not only a strong intramolecular charge transfer effect, but also a narrow electrochemical band gap and a high highest occupied molecular orbital (HOMO) energy level. The optical absorption study indicates that the PQ1 film exhibits good aggregation, which is an advantage for the charge transport between neighboring molecules. PQ1 presents p-type semiconductor properties with a high hole mobility of up to 0.12 cm^2^ V^−1^ s^−1^.

Campbell et al. created routes to increase performance for antimony selenide solar cells using inorganic hole transport layers. In this work, solar cell capacitance simulator (SCAPS) is used to interpret the effect of hole transport layers (HTL), they demonstrated the critical role of NiO and MoO_x_ in altering the energy band alignment and increasing device performance by the introduction of a high energy barrier to electrons at the rear absorber/metal interface. CSS-based Sb_2_Se_3_ solar cells with NiO HTL showed average improvements in open circuit voltage, short circuit current density and power conversion efficiency of 12%, 41%, and 42%, respectively, over the standard devices. Similarly, using a NiO HTL in TE-based Sb_2_Se_3_ devices improved open circuit voltage, short circuit current density and power conversion efficiency by 39%, 68%, and 92%, respectively.

Yang et al. reported a flexible Asymmetric organic-inorganic composite solid-state electrolyte based on PI membrane for solid-state Lithium metal batteries. This lightweight solid electrolyte is stable at a high temperature of 150°C and exhibits a wide electrochemical window of more than 6 V. Furthermore, the high ionic conductivity of the flexible solid electrolyte was 7.3 × 10^−7^ S/cm. The solid-state batteries assembled with this flexible asymmetric organic-inorganic composite solid electrolyte exhibit excellent performance at ambient temperature.

Wang et al. designed one kinds of Co_3_O_4_-doped Li_4_Ti_5_O_12_ (LTO) composites by the hydrothermal reduction and metal doping modification method. The Li_4_Ti_5_O_12_ particles attached to lamellar Co_3_O_4_ constituted a heterostructure and Co ion doped into Li_4_Ti_5_O_12_ lattice. This Co ion-doped microstructure improved the charge transportability of Li_4_Ti_5_O_12_ and inhibited the gas evolution behavior of Li_4_Ti_5_O_12_, which enhanced the lithium storage performance. It had an excellent rate performance and long cycle stability, in which the capacity reached 174.6 mA h/g, 2.2 times higher than that of Li_4_Ti_5_O_12_ at 5 A/g.

Xu et al. designed a Nano-petal nickel hydroxide on multilayered modified montmorillonite (M-MMT) using one-step hydrothermal method. This nano-petal multilayered nanostructure dominated the ion diffusion path to be shorted and the higher charge transport ability, which caused the higher specific capacitance. The results showed that in the three-electrode system, the specific capacitance of the nanocomposite with 4% M-MMT reached 1068 F/g at 1 A/g and the capacity retention rate was 70.2% after 1,000 cycles at 10 A/g, which was much higher than that of pure Ni(OH)_2_ (824 F/g at 1 A/g), indicating that the Ni(OH)_2_/M-MMT nanocomposite would be a new type of environmentally friendly energy storage supercapacitor.

## Strategies of molecular design and simulation

For better OLED performance, Liu et al. stated the strategy of molecular design in the conception of hydrogen bond interactions. By regulating the H-bond interaction, the desired properties could be obtained through restricting the rotation between different donor/acceptor moieties and inhibiting the vibrational coupling of excited states, which could obtain high luminous efficiency and color purity. Also, the multiple H-bonds interactions could further enhance horizontal orientation in amorphous organic semiconductor films and significantly increase hole and electron mobilities, which is beneficial for efficiency stability with negligible roll-off.

Sun et al. summarized a mini-review introducing strategies to improve the thermoelectric figure of merit in thermoelectric functional materials. The strategies refer to optimize the carrier concentration to improve the Seebeck coefficient, improve the steady of carrier mobility, modify the energy band to achieve expected thermoelectric performance, reduce lattice thermal conductivity, seek intrinsically low thermal conductivity thermoelectric materials and Electron-phonon decoupling.

For solar cells, where charge transport across several heterojunction interfaces is a pre-requisite for working devices. For better performance of thin-film photovoltaic devices, Jones et al. reviewed the interfaces behaviors through modelling simulation. In this review they have discussed several approaches for modelling interface processes at various length and timescales, and have split these into data-driven approaches, atomistic approaches and continuum level models. These approaches enabled better understanding of material or device performance, alongside their key limitations.

Xu et al. surveyed both theoretical calculations and experimental works to provide reference and inspiration for the rational design of multifunctional memristors, which intend to promote the increments in the memristor fabrications. As discussed in the review, the rational fabrication of memristors with Cints may give rise to remarkable enhancement in resistive switching performance with better stability and endurance, lower operation voltage, higher ON/OFF ratio, faster device speed, etc. By adjusting the Cints, suitable electric structures would be established in the metal oxides, which helps improve the performance of the electronic devices.

## Other research work relating to charge transfer properties

Some extensive research work relating to charge transfer properties were also collected in this topic. Tang et al. studied the adsorption characteristics and charge transfer kinetics of fluoride in water by different adsorbents, Zhang et al. constructed TaPP/DiDOPO conjugated flame retardant composites, which they believe the absorption properties and flame retardancy effect were enhanced due to the conjugated structure of the composites.

